# High M-MDSC Percentage as a Negative Prognostic Factor in Chronic Lymphocytic Leukaemia

**DOI:** 10.3390/cancers12092614

**Published:** 2020-09-14

**Authors:** Michał Zarobkiewicz, Wioleta Kowalska, Sylwia Chocholska, Waldemar Tomczak, Agata Szymańska, Izabela Morawska, Agnieszka Wojciechowska, Agnieszka Bojarska-Junak

**Affiliations:** 1 Department of Clinical Immunology, Medical University of Lublin, 20-093 Lublin, Poland; wioleta.kowalska@umlub.pl (W.K.); 51977@student.umlub.pl (I.M.); 2 Department of Haematooncology and Bone Marrow Transplantation, Medical University of Lublin, 20-080 Lublin, Poland; sylwia.chocholska@umlub.pl (S.C.); waldemar.tomczak@umlub.pl (W.T.); 3 Department of Clinical Transplantology, Medical University of Lublin, 20-093 Lublin, Poland; agata.szymanska@umlub.pl; 4 Faculty of Chemistry, Wroclaw University of Science and Technology, 50-370 Wroclaw, Poland; agnieszka.wojciechowska@pwr.edu.pl

**Keywords:** M-MDSC, CLL, ARG1, NOS2, IDO, TGF-β, IL-10, CD3ζ

## Abstract

**Simple Summary:**

Chronic lymphocytic leukaemia (CLL) is a malignancy of mature B cells. Tumour microenvironment is important for survival and proliferation of malignant cells. In the current study, we investigated the potential role of circulating monocytic myeloid-derived suppressor cells (M-MDSC) in CLL. We have observed an increased percentage of M-MDSC cells in CLL patients. Moreover, we have observed a close association with unfavourable prognostic markers, which suggests a potential role of M-MDSC as a prognostic factor in CLL. We have established an association between a high M-MDSC percentage on the one side and shorter time-to-treatment and overall survival on the other. Therefore, we strongly suggest to use M-MDSC percentage as another prognostic factor.

**Abstract:**

In the current study, we analysed the role and prognostic value of myeloid-derived suppressor cells (MDSC) in chronic lymphocytic leukaemia (CLL). The frequency of circulating monocytic MDSC (M-MDSC; defined as CD14^+^CD11b^+^CD15^-^HLA-DR^-/low^ cells) was assessed in correlation with clinical and laboratory parameters characterising the disease activity and patient immune status. Samples of peripheral blood from untreated CLL patients and healthy volunteers were stained with monoclonal antibodies for flow cytometry analysis. CLL patients with M-MDSC percentages above 9.35% (according to the receiver operating characteristic (ROC) analysis) had a shorter time-to-treatment and shorter survival time than the group with a lower percentage of M-MDSC. The M-MDSC percentage was higher in patients with adverse prognostic factors (i.e., 17p and 11q deletion and CD38 and ZAP-70 expression). A high M-MDSC percentage was linked to significantly lower expression of the CD3ζ in T cells. Furthermore, an analysis of immune regulatory molecules (arginase 1 (ARG1), nitric oxide synthase (NOS2), indoleamine 2,3-dioxygenase (IDO), transforming growth factor beta (TGF-β), and interleukin (IL)-10) was performed. By the means of flow cytometry and RT-qPCR, we showed an overexpression of three of them in M-MDSC of CLL patients. M-MDSC cells seem to be an important factor in the immunosuppressive microenvironment of CLL and seem to be a good and novel prognostic factor

## 1. Introduction

Myeloid-derived suppressor cells (MDSC) represent a heterogeneous population of immature myeloid cells associated with cancer and other pathological conditions [1,2]. Based on their phenotypic and morphological features, MDSC can be divided into two subsets: polymorphonuclear (PMN-MDSC, expressing CD15) and monocytic (M-MDSC, expressing surface CD14) [1,3]. MDSC are able to suppress different immune cells, e.g., T, natural killer (NK), and B lymphocytes [2,4]. Simultaneously, they are the enhancers of other immunosuppressive cells, e.g., regulatory T (Treg) and B cells (Breg) [5]. MDSC produce a number of molecules that are involved in immune suppression [2,5]. For instance, they secrete interleukin (IL)-10 and transforming growth factor beta (TGF-β), two potent anti-inflammatory and immunosuppressive cytokines [5,6,7]. MDSC also generate nitric oxide (NO) by promoting the expression of inducible nitric oxide synthase (NOS2/iNOS) [5]. In tumour biology, NO is usually perceived as an immunosuppressive molecule that inhibits T-cell activation and the antitumour immune response [8,9]. In addition, the activation of MDSC induces the expression of arginase 1 (ARG1) and indoleamine 2,3-dioxygenase (IDO), which catabolise L-arginine and tryptophan, respectively [1,2]. An accumulation of MDSC has been demonstrated in many types of human solid tumours. MDSC levels were found to correlate with tumour progression, metastasis, and recurrence [10]. Furthermore, a relationship between the frequency of circulating MDSC and clinical responses to radio-, chemo-, and immunotherapy was found [11,12]. The importance of these cell types in haematopoietic malignancies has only recently gained stronger attention. The increased frequency of MDSC was found in multiple myeloma [13]. They are also expanded in the peripheral blood of DLBCL (diffuse large B cell lymphoma) patients with a more aggressive clinical course [14]. Knowledge about M-MDSC in indolent lymphomas is currently severely limited [15]. Nevertheless, the percentage of M-MDSC in the peripheral blood of various indolent lymphomas (mostly follicular lymphomas) is significantly higher when compared to healthy controls, while being significantly lower than in more aggressive lymphomas [16,17]. In addition, a monocytic subset of MDSC (CD14^+^HLA-DR^low^) is increased in CLL (chronic lymphocytic leukaemia) patients and has a capacity to inhibit T cell activation, as well as induce Treg via IDO [18]. Concurrently, no difference was observed in mycosis fungoides [19] and Waldenström macroglobulinemia [17].

Many issues in CLL development and progression are still unclear. Likewise, the role of MDSC in CLL immunopathogenesis remains undefined. CLL is a malignancy of mature B cells, which survival and proliferation depend on interactions with nonmalignant cells in the tumour microenvironment [14,20,21]. In the current study, we investigated the potential role of MDSC in CLL patients by analysing the frequency of circulating M-MDSC in correlation with clinical and laboratory parameters characterising the disease activity and patient immune status. Moreover, an analysis of immune regulatory molecules related to the MDSC functions (such as ARG1, NOS2, IDO, TGF-β1, and IL-10) was performed.

## 2. Results

### 2.1. The Percentage of M-MDSC Is Increased in the Peripheral Blood of CLL Patients and Correlates with The Rai Stage

**Figure 1:**
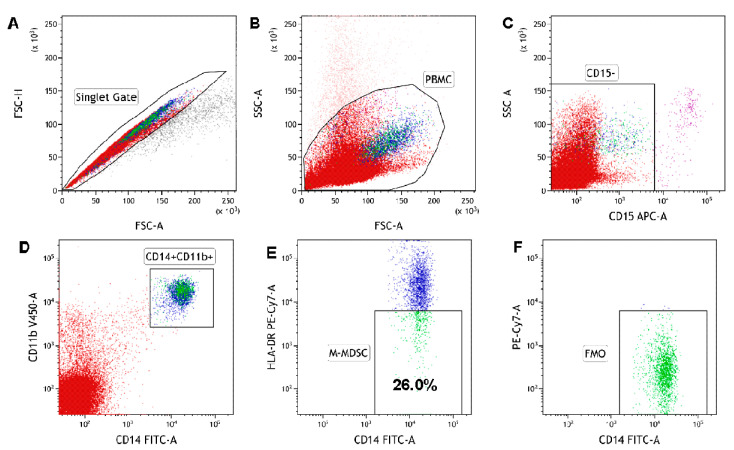
The identification of the monocytic myeloid-derived suppressor cell (M-MDSC) population in peripheral blood mononuclear cells (PBMC). PBMC are stained and acquired using a flow cytometer. M-MDSC were identified with the following gating strategy. (**A**) A combination of forward scatter area (FSC-A) and forward scatter height (FSC-H) was utilized to exclude doublets. (**B**) After gating for singlets, mononuclear cells were selected based on their side scatter/forward scatter (SSC/FSC) properties. (**C**) Gate on the CD15-negative (CD15^−^) cell population in CD15 APC vs. SSC-A dot plot. (**D**) Within the CD15^−^ population, CD14^+^CD11b^+^ cells were gated in CD14 FITC vs. the CD11b V450 dot plot. (**E**) Selected CD14^+^CD11b^+^ cells were analysed for HLA-DR expression. The final dot plots (CD14 FITC vs. HLA-DR PE-Cy7) indicate CD14^+^CD11b^+^CD15^−^HLA-DR^−/low^ cells (M-MDSC). The gate for HLA-DR^-/low^ cells was set based on the FMO (fluorescence minus one) control. The plot (**F**) is an FMO control for HLA-DR, stained with all markers in the panel, except for HLA-DR. Data were analysed using Kaluza 2.1.1 software.

**Figure 2:**
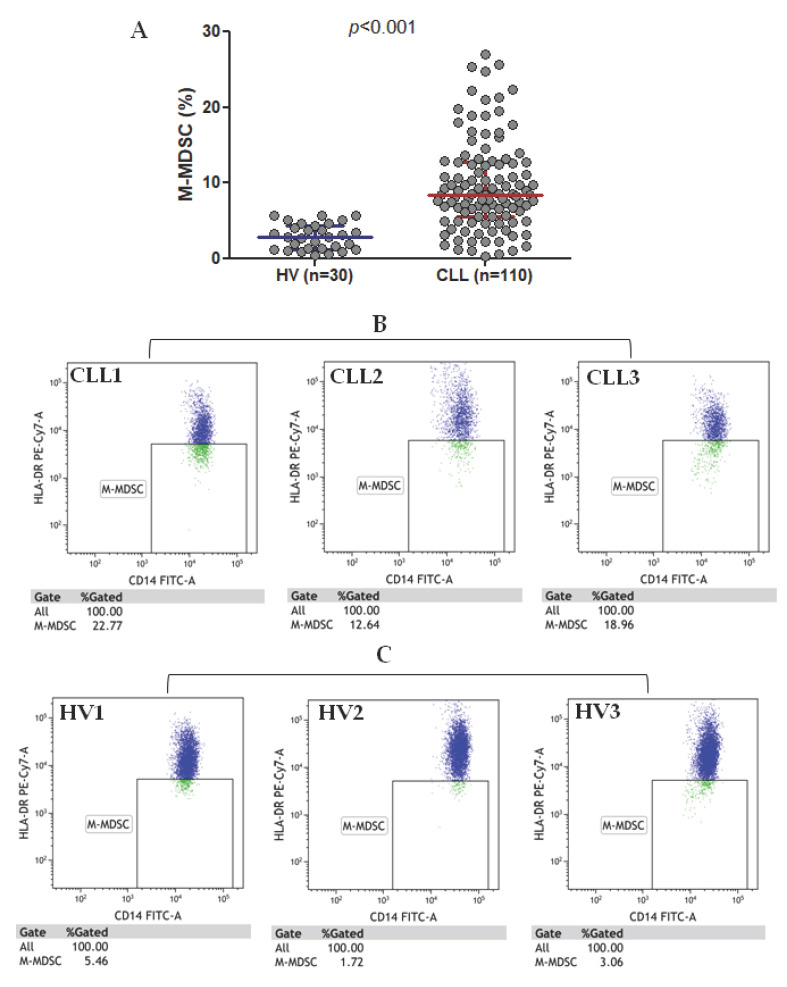
M-MDSC percentage in chronic lymphocytic leukaemia (CLL) patients and healthy volunteers (HVs) (**A**) and representative flow cytometry data (dot plots) (**B**,**C**). Three CLL cases (CLL1-CLL3) and three healthy volunteers (HV1-HV3) are shown.

M-MDSC in the current study are defined as CD14^+^CD11b^+^CD15^-^HLA-DR^-/low^ cells; the gating strategy is presented in Figure 1. M-MDSC are significantly expanded in CLL patients compared to healthy volunteers (HVs) (median interquartile range (IQR), 8.41 (5.55–12.78%) vs. 2.82 (1.28–4.42%), *p* < 0.01; Figure 2A). Representative dot plots from three CLL patients and three healthy donors with different M-MDSC percentages are shown in Figure 2B,C. 

**Figure 3:**
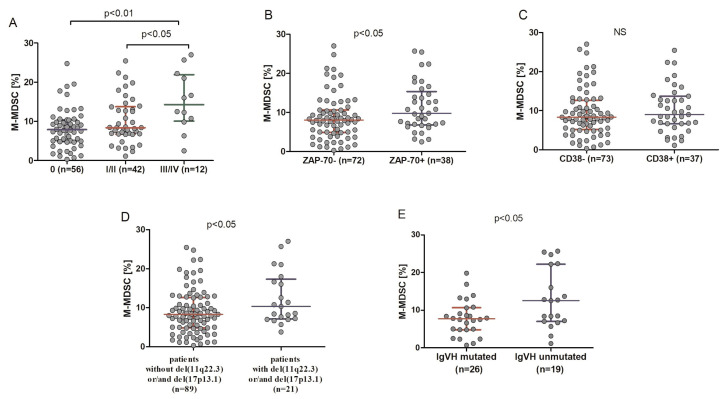
Percentage of M-MDSC in CLL patients in different disease stages (**A**), in ZAP-70-positive and ZAP-70-negative CLL patients (**B**), and CD38-positive and CD38-negative ones (**C**). M-MDSC frequency in CLL patients carrying the 11q22.3 and/or the 17p13.1 deletion and patients without these genetic aberrations (**D**). M-MDSC percentages in unmutated and mutated CLL cases (**E**). The central line shows the median and “whiskers” represent the interquartile range (IQR). IGHV: immunoglobulin heavy chain variable.

A high diversity of M-MDSC percentages was noted in CLL patients, with significantly lower values in patients at stage 0 (median (IQR), 7.88 (4.86–10.36%)) as compared to those in the stages I and II (median (IQR), 8.39 (6.73–13.74%)) and III and IV (median (IQR), 14.26 (10.07–21.93%)), according to the Rai stages (Figure 3A). Concurrently, very low interindividual variability was observed in HVs. Among CLL patients, there was a weak positive correlation between the percentage of M-MDSC and the white blood cells (WBC) count (r = 0.262, *p* < 0.05) and peripheral blood (PB) lymphocyte count (r = 0.270, *p* < 0.05); no correlations were identified for the platelet counts, haemoglobin concentration, serum lactate dehydrogenase (LDH), and β2-microglobulin levels or the ages of the patients.

### 2.2. M-MDSC Percentage Is Higher in Patients from High-Risk Groups

The percentage of M-MDSC was significantly higher in ZAP-70-positive patients (median (IQR), 9.79 (6.82–15.34%)) compared with ZAP-70-negative ones (median (IQR), 8.07 (4.85–10.61%)) (*p* < 0.05) (Figure 3B). No significant difference in the percentage of M-MDSC between CD38-positive (median (IQR), 8.98 (6.65–13.68%)) patients and CD38-negative ones (median (IQR), 8.34 (5.13–12.65%)) was noted (Figure 3C). On the other hand, as shown in Figure 3D, there was a significant difference in the median percentage of M-MDSC between patients carrying the 11q22.3 and/or the 17p13.1 deletion (median (IQR), 10.30 (7.12–17.30%)) and patients without these genetic aberrations (median (IQR), 8.30 (4.81–12.59%)) (*p* < 0.05). In CLL patients with mutated immunoglobulin heavy chain variable (IGHV) genes, the M-MDSC percentage was lower (median (IQR), 7.68 (4.81–10.66%)) than in those with unmutated IGHV (median (IQR), 12.54 (7.01–22.22%)) (*p* < 0.05) (Figure 3E). 

### 2.3. High M-MDSC Percentage Is a Negative Prognostic Marker for Time-to-Treatment and Survival Time

**Figure 4:**
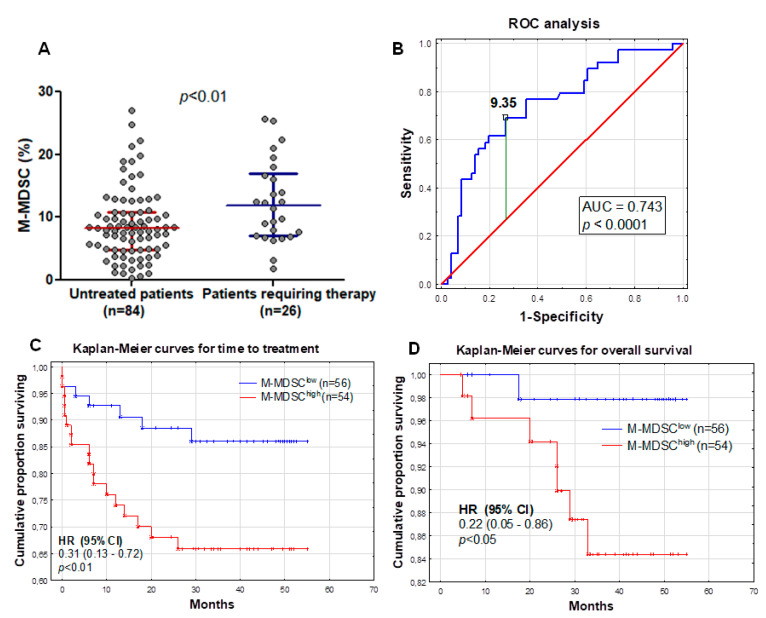
M-MDSC percentages in two groups of patients—requiring and not-requiring therapy during the follow-up period (**A**). Receiver operating characteristic (ROC) and area under the curve (AUC) were used to evaluate the sensitivity and specificity and to calculate the most significant cut-off value of M-MDSC percentages that best distinguished ZAP-70-positive and ZAP-70-negative cases (**B**). Kaplan-Meier curves comparing the length of time-to-treatment (TTT) among CLL patients based on the MDSC cut-off value of 9.35% (**C**). Similar Kaplan-Meier curves comparing the overall survival of CLL patients (**D**). HR, hazard ratio and CI, confidence interval.

To assess, the prognostic significance of M-MDSC in CLL patients was observed for a prolonged time, with a median follow-up time of 38 months (range 1–55 months). Time-to-treatment (TTT) was defined as the period from initial diagnosis to the initialization of treatment. During that period, 23.6% patients (*n* = 23) required chemotherapy and 7.3% died (*n* = 8); the median TTT was 31 months. Patients who required therapy during the follow-up period had significantly higher M-MDSC percentages than those without the need for treatment (median (IQR), 11.85 (7.0–16.98%) vs. 8.23 (4.85–10.81%), *p* < 0.01; Figure 4A). 

The analysis of the receiver operating characteristic (ROC) curves and area under the curve (AUC) revealed that M-MDSC percentages could be a potential CLL clinical biomarker. Based on the ROC analysis, we determined that the optimum M-MDSC threshold associated with ZAP-70 above 20% was 9.35% (AUC, 0.743; sensitivity, 69.2%; specificity, 73.2%; and 95% confidence interval (CI), 0.646–0.840; *p* < 0.0001; Figure 4B). Using 9.35% as a cut-off value, we divided our cohort into two groups: M-MDSC^low^ (less than 9.35% of M-MDSC; *n* = 56) and M-MDSC^high^ (9.35% or more of M-MDSC; *n* = 54) groups. M-MDSC^low^ and M-MDSC^high^ patient characteristics at the time of CLL diagnosis are summarized in Table 1. As expected, M-MDSC^low^ patients were more often at Rai stage 0 (58.9%). There was no significant difference between the two groups in terms of age, platelets count, β2-microglobulin, and haemoglobin levels. However, there was a significant difference between the groups in WBC count (*p* < 0.05) and the number of circulating lymphocytes (*p* < 0.01).

There was a significant association between M-MDSC percentages above 9.35% and a shorter TTT (hazard ratio (HR) = 0.31; 95% CI 0.13–0.72; *p* < 0.01; median TTT: 26 months vs. 40 months in M-MDSC^low^) (Figure 4C). In the univariate analysis, we have shown that the M-MDSC percentage was associated with a shorter TTT in CLL patients (Table 2). Next, we determined whether the percentage of M-MDSC retained the prognostic significance in the multivariate analysis. We performed the univariate and multivariate analyses by Cox proportional hazard regression to detect the independent prognostic factors related to the TTT of CLL patients. We included the classical risk factors like age; genetic aberrations (del(17p13.1) and del(11q22.3)); IGHV mutational status; and CD38, ZAP-70, and LDH, as well as β2M, levels (Table 2). In univariate analyses, ZAP-70 ≥ 20%, CD38 ≥ 30%, and β2M ≥ 3.5 mg/L; the presence of unfavourable cytogenetic abnormalities; and M-MDSC ≥ 9.35% were significantly associated with shorter TTT (Table 2). Subsequently, these five parameters were included in multivariate Cox regression analyses. Finally, ZAP-70, β2M, the genetic aberrations, and M-MDSC were independent predictors of shorter TTT (Table 2). 

We found a significant association between the percentage of M-MDSC above 9.35% and the overall survival (OS) (HR = 0.22; 95% CI 0.05–0.86; *p* < 0.05). The M-MDSC^high^ group showed significantly shorter survival times compared with the M-MDSC^low^ group (median OS: 33 months vs. 45 months) (Figure 4D). Similar to the TTT, further univariate and multivariate analyses were performed. A higher β2M level, the presence of unfavourable cytogenetic abnormalities, and a high ZAP-70 expression were significantly associated with a worse OS in the univariate analysis (Table 3). The IGHV mutational status was accessible for only 45 participants of this study, and all of them survived the follow-up period; thus, assessment of the OS was not possible. A further multivariate analysis did not confirm an independent prognostic effect of the M-MDSC percentage on the OS, while the opposite was noted for the β2M level (Table 3). 

### 2.4. IDO, ARG1, NOS2, IL-10, and TGF-Β1 Are Overexpressed by CLL-Derived M-MDSC

**Figure 5:**
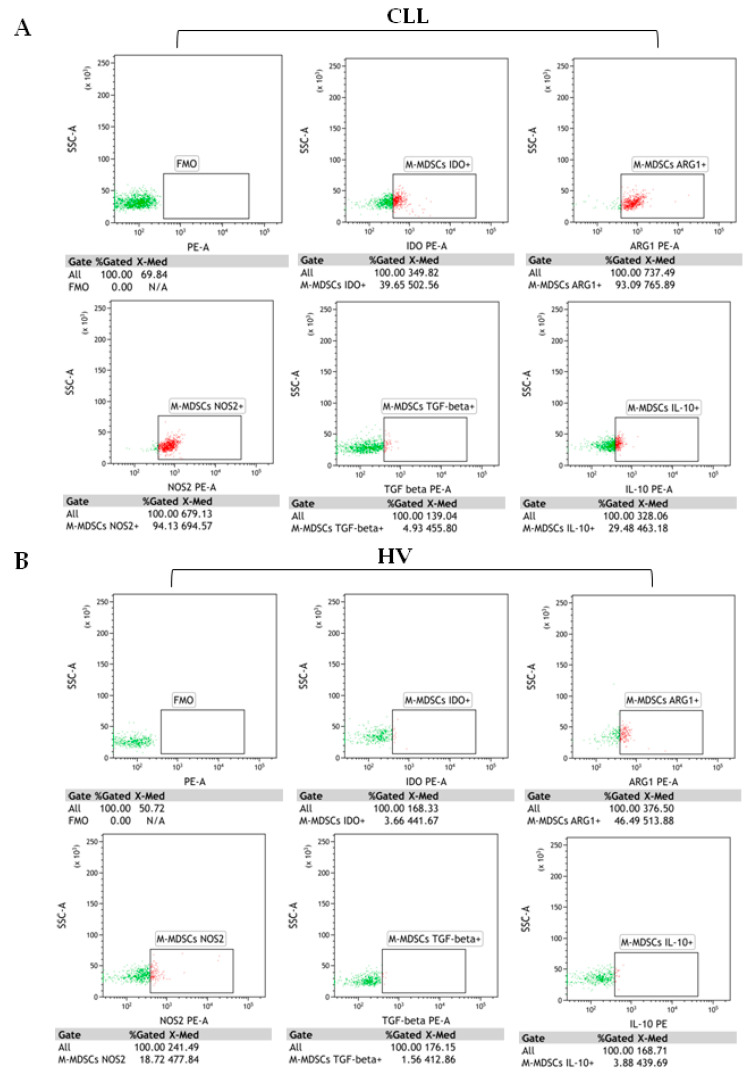
M-MDSC (CD14^+^CD11b^+^CD15^-^HLA-DR^-/low^) with intracellular indoleamine 2,3-dioxygenase (IDO), arginase 1(ARG1), nitric oxide synthase (NOS2), transforming growth factor beta 1 (TGF-β1), and interleukin (IL)-10 expressions. Representative flow cytometry dot plots from (**A**) CLL cases and (**B**) healthy volunteers. Immune regulatory proteins were evaluated by the intracellular staining of M-MDSC. The dot plots PE vs. SSC are the FMO (fluorescence minus one) controls, stained with all markers in the panel, except for IDO, ARG1, NOS2, IL-10, or TGF-β1. FMO controls were used to control for the gating. The gating strategy to identify M-MDSC is shown in Figure 1. The results are expressed as the percentage of M-MDSC with intracellular IDO, ARG1, NOS2, IL-10, or TGF-β1 expressions. Under the individual dot plots, there are also MFI (median fluorescence intensity) values. Data were analysed using Kaluza 2.1.1 software.

In addition to surface markers, we analysed the expression of immune regulatory molecules related to the MDSC functions (ARG1, NOS2, IDO, TGF-β1, and IL-10). The analysis was performed on PBMC freshly isolated from the peripheral blood (without culture or MDSC stimulation). All five proteins were significantly overexpressed in CLL-derived M-MDSC (Table 4). Representative dot plots from CLL patient and healthy donors are shown in Figure 5A,B. 

**Figure 6:**
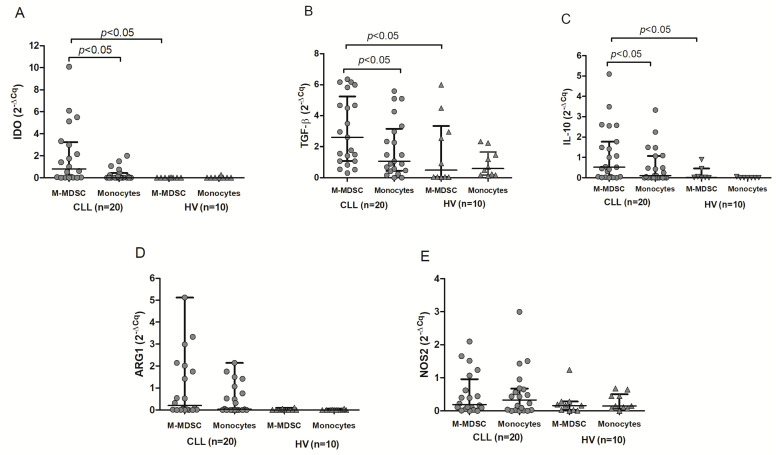
Quantitative expression of IDO (**A**), TGF-β (**B**), **IL-**10 (**C**), ARG1 (**D**), and NOS2 (**E**) mRNA. RT-qPCR was performed on RNA samples isolated from HLA-DR^-/low^ (M-MDSC) and HLA-DR^high^ (monocytes) cells obtained from CLL patients (*n* = 20) or healthy volunteers (HV; *n* = 10).

RT-qPCR was used to quantify the levels of mRNA expression for regulatory molecules in purified M-MDSC. RT-qPCR confirmed the presence of IDO, ARG1, NOS2, TGF-β1, and IL-10 transcripts in M-MDSC. IDO, TGF-β1, and IL-10 were also significantly overexpressed in CLL compared to HV M-MDSC (*p* < 0.05). Consistent with flow cytometry, IDO, TGF-β1, and IL-10 mRNA levels were significantly higher in the HLA-DR^-/low^ (M-MDSC) fraction as compared to the HLA-DR^high^ (monocytes) in CLL patients (*p*<0.05) (Figure 6A–C). Similarly, there was no significant difference between the two fractions in terms of ARG1 (Figure 6D) and NOS2 (Figure 6E) mRNA expression (*p* > 0.05).

### 2.5. The CD3 Ζ Chain (CD247) Expression in T Lymphocytes Is Significantly Lower in the M-MDSC^high^ Group

**Figure 7:**
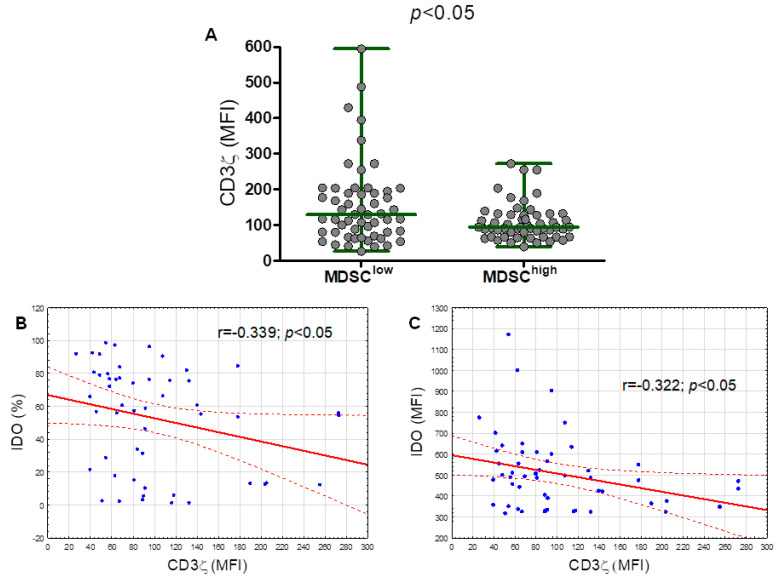
The ζ chain (CD247) expression (based off the mean fluorescence intensity, MFI) in T lymphocytes from the M-MDSC^low^ (less than 9.35% of M-MDSC) and M-MDSC^high^ (9.35% or more of M-MDSC) groups (**A**). Correlation of the CD3ζ expression with the percentage of M-MDSC with the intracellular IDO expression in the peripheral blood of CLL patients (**B**). Correlation of the CD3ζ expression with the IDO expression (indicated by the MFI) in M-MDSC (**C**).

The level of CD3ζ expression determined by median fluorescence intensity (MFI) was significantly (*p* < 0.050) higher in the M-MDSC^low^ group (median (IQR), 129.9 (80.04–195.30) MFI) compared with the M-MDSC^high^ (median (IQR), 94.84 (67.11–129.90) MFI) (Figure 7A). Moreover, the analysis of T lymphocytes in terms of the ζ chain expression found a weak significant inverse correlation between the M-MDSC percentage with an intracellular IDO expression and the intracellular CD3ζ expression in T cells (Figure 7B). The CD3ζ expression inversely correlated with the IDO expression (based on the MFI) (Figure 7C). No correlation between the CD3ζ expression and ARG1, NOS2, TGF-β1, and IL-10 expressions in M-MDSC was detected.

## 3. Discussion

An important part of the CLL pathogenesis is its microenvironment with MDSC as one of its major components. It is the microenvironment that promotes the survival and expansion of neoplastic B cells and determines the course of the disease [20,22]. Still, the importance of MDSC in haematological malignancies has not gained the desired attention until recently [14]. In the current paper, we report the accumulation of M-MDSC and their prognostic values in CLL patients. 

In accordance with previous studies [17,23,24], we found an increased frequency of M-MDSC in CLL patients in comparison to healthy controls. M-MDSC are also expanded in the peripheral blood of freshly diagnosed patients with Hodgkin’s lymphoma, follicular lymphoma, diffuse large B-cell lymphoma, and other haematological malignancies [18]. Consistent with our study, Liu et al. revealed that M-MDSC cells are associated with the CLL clinical stage [25]. In the present study, the M-MDSC level was also significantly higher in ZAP-70-positive cases, which is in contrast to Jitschin et al. [18]. The observed discrepancies may result from a diverse set of markers used to identify the M-MDSC, a different gating strategy, or a smaller study group [1,26]. We have also found a significant difference in M-MDSC percentages between patients carrying the 11q22.3 and/or the 17p13.1 deletion and patients without these genetic aberrations. Similar to Liu et al. [25], we have noted that M-MDSC are associated with IGHV mutational status—the frequency of M-MDSC was significantly higher in the IGHV-unmutated (U-CLL) patients. 

The mechanism of M-MDSC expansion in humans is not fully clear. M-MDSC are probably recruited from the bone marrow and then released into the circulation where they can mix with circulating malignant cells of haematopoietic origin. Liu et al. demonstrated that CD14^+^HLA-DR^low/−^ MDSC positively correlated with CD19^+^CD5^+^ cells, contributing to disease progression [25]. MDSC expansion and survival is at least partially dependent on tumour-produced growth factors [27]. Moreover, CLL-derived exosomes (containing miR-155) have significant potential to drive monocytes into M-MDSC [28]. Ultimately, cocultures of monocytes with CLL cells lead to the induction of M-MDSC [18]. This interdependence between MDSC and cancerous cells would explain the correlations between disease progression and M-MDSC percentage in peripheral blood in a number of haematological malignancies.

In the current study, patients who required therapy had significantly higher M-MDSC percentages at the time of diagnosis than in those who received no therapy during follow-up. This is concordant with data reported by Jitschin et al., Gustafsson et al., and Zahran et al. [18,23,24]. Gustafsson et al. observed a significantly shorter time-to-progression in CLL patients with high M-MDSC counts [23]. Similarly, a correlation between the M-MDSC count and event-free-survival was observed in diffuse large B cell lymphoma patients [29]. The percentage of M-MDSC rises also with the progression of multiple myeloma [30]. It is a unique and important finding of our study that there was an association between a high M-MDSC number and shorter time-to-treatment. Patients with high M-MDSC had a significantly shorter TTT. Furthermore, the multivariate analysis confirmed that TTT is significantly and independently affected not only by ZAP-70 expression or the presence of unfavourable cytogenetic abnormalities but, also, by the baseline M-MDSC percentage. This is an important novelty of the current study. Moreover, it backs up the significance of MDSC as a new prognostic factor. To our knowledge, this is the first such multivariable model adapting traditional prognostic markers and M-MDSC in CLL. 

In our study, a high percentage of M-MDSC was also associated with shorter survival times. Similarly, Zahran et al. reported a significantly higher overall survival time in CLL patients with low M-MDSC levels (<25%) compared to those with high MDSC levels (>25%) [24]. A variety of cut-offs were used to define a case as being M-MDSC^high^—25% [24], 40% [25], and 9.35%—in our study. Moreover, different methods are also used for M-MDSC identification. Generally, M-MDSC are characterised as CD14+HLA-DR-/low [23,24,25,30]. In our study, M-MDSC were defined as CD14^+^CD11b^+^CD15^−^HLA-DR^−/low^ cells. This is in-line with recent recommendations by Bronte et al. [1]. To the best of our knowledge, we have used the optimal four-colour antibody panel in our research [26]. The results of various groups are not fully comparable, owing to the different compositions of patient populations and the diversity in cut-off values defining “M-MDSC^high^”. In our study, ROC curves were used to determine the most significant cut-off values of M-MDSC. Finally, our study was performed on a large group of CLL patients (*n* = 110), with a long follow-up time (median: 38 months).

The primary functional characteristic of M-MDSC is the capacity to suppress immune cells, predominantly T lymphocytes and, to a lesser degree, B lymphocytes and NK cells [15,31]. M-MDSC suppression is mediated by various mechanisms, such as the upregulation of ARG1 expression, iNOS/NOS2, and IDO. M-MDSC can also express immunosuppressive cytokines such as TGF-β or IL-10 [1,31]. These cytokines may create an autocrine feed-forward loop that promotes M-MDSC accumulation [1]. In the current study, the analysis of molecules indicating M-MDSC functional activity was focused on cells directly isolated from peripheral blood (without culture and in vitro M-MDSC stimulation, thus resembling as close as possible the real in vivo conditions [32]). The immune regulatory molecule expression was determined by RT-qPCR and flow cytometry. RT-qPCR and flow cytometry confirmed the presence of ARG1, NOS2, IDO, TGF-β, and IL-10 both at the protein and mRNA levels in M-MDSC. Similar to our results, Jitschin et al. observed a significant upregulation of IDO within CLL-derived M-MDSC and monocytes cocultured with CLL cells [18]. IDO is at least partially responsible for the suppressive potential of M-MDSC [18]. It is an important enzyme that degrades L-tryptophan. The lack of tryptophan and production of kynurenine results in T-cell anergy, cell cycle arrest, and the promotion of CD4 T lymphocyte conversion to immunosuppressive Tregs [12,31]. 

The CD3ζ chain is a key molecule for the transduction of stimulatory signals through the T-cell receptor (TCR). As noted by Whiteside TL, scoring lymphocytes for CD3ζ expression by MFI provides a quantitative measurement for a population of cells in suspensions [33]. We have shown that T-cell ζ-chain expressions (MFI) in CLL patients were downregulated in the M-MDSC^high^ group compared with the M-MDSC^low^ group. Even though, in the current study, all marked immune regulatory molecules were significantly overexpressed in CLL-derived M-MDSC, only IDO seems to be significantly involved in M-MDSC-mediated T-cell suppression (manifested by a decrease in CD3ζ chain expression). High intracellular IDO expression in M-MDSC inversely correlated with the CD3ζ expression. Our results are in-line with the study by Jitschin et al., who reported that the MDSC-mediated modulation of T cells could be attributed to their increased IDO activity [18]. It is an important limitation of the current study that no experimental approach was used for the direct assessment of M-MDSC influence on CD3ζ expression. We hope to address this issue in the near future.

IL-10 and TGF-β produced by M-MDSC cells promote the expansion of T-regulatory cells [34,35]. IL-10 and TGF-β can also promote the build-up of M-MDSC cells [36,37]. TGF-β, similarly, drives the expansion of M-MDSC themselves and the establishment of the overall immunosuppressive microenvironment [34]. Moreover, MDSC promotes the expansion of another immunosuppressive subset—regulatory B cells (Breg) [38]. It seems that, in CLL patients, circulating M-MDSC are a rich source of TGF-β and IL-10. In our previous study, we observed a close association of TGF-β+ M-MDSC with unfavourable prognostic markers (i.e., ZAP-70, CD38, and 11q and 17p deletion) [39]. Similarly, Sato et al. showed that the increased frequencies of IL-10-producing MDSC in non-Hodgkin’s lymphoma patients were associated with decreased NK cells in the peripheral blood [40]. NO synthesis and iNOS activity are important for the M-MDSC-driven suppression of NK-mediated cytotoxicity [41]. Finally, we have also observed increased frequencies of ARG1- and NOS2-positive M-MDSC cells in CLL patients. Cells expressing either of those enzymes can downregulate the expression of CD3ζ and reduce the proliferation in T lymphocytes by depleting the available extracellular L-arginine [35].

NOS2, ARG, IDO, TGF-β1, and IL-10 may also be expressed in monocytes/macrophages. The flow cytometry analysis of those molecules in MDSC should be compared to monocytes/macrophages as an additional control [35]. In our study, IDO, ARG1, NOS2, IDO, TGF-β1, and IL-10 expressions in M-MDSC and monocytes were compared by both flow cytometry and RT-qPCR. M-MDSC had high intracellular IDO, IL-10, and TGF-β quantities, which is in-line with Jitschin et al. [18]. Although we have noted high levels of ARG1 and NOS2 in CLL M-MDSC, those enzymes were expressed at similar levels in HLA-DR^high^ monocytes. Interestingly, recent data suggest that ARG1 is not constitutively expressed in MDSC nor required for MDSC-mediated inhibition [42]. However, the differentiation of M1 and M2 monocytes/macrophages is regulated by cardinal genes that include iNOS and ARG1, among others [43]. Moreover, the different immunosuppressive mechanisms do not have to work simultaneously [44]. Marvel et al. suggested that, at a given time, there is a dominant suppression mechanism used by MDSC [44]. Moreover, this mechanism may change as the disease progresses. Our data suggest that IDO, IL-10, and TGF-β are responsible for M-MDSC-mediated immunosuppression in CLL patients. 

## 4. Materials and Methods 

### 4.1. Patients and Samples

One hundred and ten patients newly diagnosed with CLL were recruited in the Department of Haematooncology and Bone Marrow Transplantation of the Medical University of Lublin (Lublin, Poland) in the period between January 2014 and June 2019. The diagnosis was based on criteria from the International Workshop on Chronic Lymphocytic Leukemia (IWCLL) [45]. A sample of peripheral blood (PB) was collected at the time of initial diagnosis, as always prior to any anticancer therapy. At the time of recruitment, the clinical stage was determined according to the Rai classification system [46]. The majority (*n* = 56) were in stage 0, nearly one-fourth (*n* = 25) were in stage I, 17 patients were in stage II, 7 patients were in stage III, and 5 patients were in stage IV. The basal characteristics of the patients are presented in Table 2. The control group consisted of 30 healthy volunteers (HV; 16 females and 14 males, aged 36–72 years; median, 58 years). Both from CLL patients and HV, PB samples were collected into EDTA-containing tubes and processed immediately. The separation of peripheral blood mononuclear cells (PBMC) was conducted by density gradient centrifugation using Gradisol L (Cat No.: 9003.1, Aqua-Med, Łódź, Poland). Samples were centrifuged for 25 minutes at 400 × g at room temperature. Immediately afterwards, interphase cells were collected, washed twice, and resuspended in phosphate-buffered saline (PBS).

This study was approved by the Ethics Committee of the Medical University of Lublin (No. KE-0254/107/2013 and KE-0254/49/2016). Written informed consent was obtained from all patients with respect to the use of their blood for scientific purposes. 

### 4.2. Detection of M-MDSC

Flow cytometry analysis of M-MDSC (defined as CD14^+^CD11b^+^CD15^-^HLA-DR^-/low^ cells) was performed on freshly isolated PBMC. Samples were stained with a combination of fluorescent-labelled monoclonal antibodies: mouse anti-human CD14 FITC (Clone MφP9, Cat No.: 347493), mouse anti-human CD11b V450 (Clone ICRF44, Cat No.: 560481), mouse anti-human HLA-DR PE-Cy7 (Clone L243, Cat No.: 335795), and mouse anti-human CD15 APC (Clone HI98, Cat No.: 551376). All the monoclonal antibodies were purchased from BD Biosciences (Franklin Lakes, NJ, USA). Cells were incubated for 20 min at room temperature (RT). 

### 4.3. Analysis of Intracellular IDO, Arg1, NOS2, IL-10, or TGF-Β1 Expressions 

Analysis of intracellular IDO, Arg1, NOS2, IL-10, or TGF-β1 expressions by M-MDSC was performed on fresh PBMC from 60 CLL patients and 20 HV. PBMC were stained with monoclonal antibodies against cell-surface markers: CD14, CD11b, HLA-DR, and CD15 (as described above). Following membrane staining, cells were fixed with Cytofix/Cytoperm and permeabilized with perm/wash buffer (BD Biosciences, Cat No.: 554714), according to the manufacturer’s protocol. Cells were then intracellularly stained (20 min at RT) with mouse anti-human indoleamine 2,3-dioxygenase/IDO PE-conjugated antibody (Clone 700838, Cat No.: IC6030P), mouse anti-human arginase 1/ARG1 PE-conjugated antibody (Clone 658922, Cat No.: IC8026P) (R&D Systems, Inc., Minneapolis, MN, USA), PE rat anti-human IL-10 antibody (Clone JES3-19F1, Cat No.: 506804), PE mouse anti-human LAP (TGF-β1) antibody (Clone TW4-2F8, Cat No.: 349604) (BioLegend, San Diego, CA, USA), or mouse anti-human NOS2 antibody (C-11) PE (sc-7271, Santa Cruz Biotechnology, Inc., Santa Cruz, CA, USA). Fluorescence minus one (FMO) controls were used to control for the gating.

### 4.4. Flow Cytometry Analysis of M-MDSC

Directly after staining, samples were analysed on BD FACSCanto II (detailed configuration in Table S1) with BD FACSDiva Software (BD Biosciences, Franklin Lakes, NJ, USA). At least 100,000 events were acquired and analysed for each tube. Kaluza 2.1.1 software (Beckman Coulter, Miami, FL, USA) was used for the data analysis. Forward (FSC) and side scatter (SSC) were used to gate lymphocytes. An example of the detailed gating strategy used in the current study is shown in Figure 1A–E. Wherever a percentage of M-MDSC is mentioned, it is a percentage among the peripheral blood CD14^+^CD11b^+^ cells. (Figure 1E). Additionally, in the experiment, the percentage of M-MDSC (CD14^+^CD11b^+^CD15^-^HLA-DR^-/low^) cells with IDO, ARG1, NOS2, IL-10, or TGF-β expression was determined (Figure 5). Moreover, within the M-MDSC population, immune regulatory molecules were quantified regarding their median fluorescence intensity (MFI). Data were normalized to the FMO control and presented as ΔMFI. ΔMFI is the difference between the MFI of the specimen stained for IDO, ARG1, NOS2, IL-10, or TGF-β and the MFI of the same channel in FMO control (e.g., ΔMFI = MFI IDO (PE) – MFI FMO (PE)).

### 4.5. Flow Cytometry Sorting of M-MDSC Cells For Reverse Transcription-Quantitative Polymerase Chain Reaction (RT-Qpcr)

**Figure 8:**
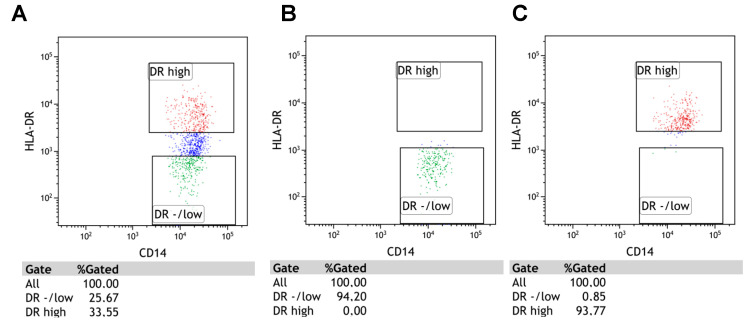
The results of cell sorting on BD FACSAria IIu. Panel (**A**) shows the pre-sort total population of CD14^+^CD11b^+^CD15^-^. HLA-DR^high^ (monocytes) and HLA-DR^low^ (M-MDSC) cells were sorted with high purity. Panels (**B**,**C**) show the post-sort conditions.

In 20 CLL cases and 10 healthy volunteers, the CD14^+^CD11b^+^CD15^-^ monocytes were sorted into HLA-DR^-/low^ (M-MDSC) and HLA-DR^high^ (monocytes) fractions. BD FACSAria IIu (detailed configuration in Table S1) (BD Biosciences, Franklin Lakes, NJ, USA) was used for cell sorting. In this case, the samples were labelled with the following monoclonal antibodies: mouse anti-human CD14 FITC (Clone MφP9, Cat No.: 347493), mouse anti-human CD11b PE-Cy7 (Clone ICRF44, Cat No.: 557743), mouse anti-human HLA-DR PE (Clone L243, Cat No.: 307606), and mouse anti-human CD15 APC (Clone HI98, Cat No.:551376) (all from BD Biosciences, Franklin Lakes, NJ, USA; only HLA-DR PE was from BioLegend, San Diego, CA, USA). Cells were incubated for 20 min at room temperature and then washed with PBS; following which, the HLA-DR^-/low^ and HLA-DR^high^ populations were sorted (Figure 8A–C).

### 4.6. RNA Preparation and RT-Qpcr for IDO, ARG1, NOS2, IL-10, and TGF-Β1

Purified HLA-DR^−/low^ (M-MDSC) and HLA-DR^high^ (monocytes) fractions were used for RNA isolation. Total RNA was isolated using the QIAamp RNA Blood Mini Kit (Cat No.: 52304; Qiagen, Inc., Valencia, CA, USA). RNA quantity and quality were measured using a BioSpec nano-spectrophotometer (Shimadzu Biotech, Kyoto, Japan). RNA was transcribed into cDNA using the QuantiTect Reverse Transcription kit (Cat No.: 205311; Qiagen, Inc., Valencia, CA, USA), according to the manufacturer’s protocol. RT-qPCR was performed using TaqMan Gene Expression Assays (Thermo Fisher Scientific, Applied Biosystems, Inc., Waltham, MA, USA; assay ID: IL-10 (Hs00961622_m1), IDO (Hs00984148_m1), ARG1 (Hs00163660_m1), and NOS2 (Hs01075529_m1), TGFB1 (Hs00998133_m1) and TaqMan Gene Expression Master Mix (Cat No.: 4369016)). β-actin was used as the internal control (Human ACTB (Beta Actin) Endogenous Control, Cat No.: 4310881E; Thermo Fisher Scientific, Applied Biosystems, Inc., Waltham, MA, USA). RT-qPCR reactions were run for 40 cycles using universal cycling conditions (95 °C for 10 min, followed by 40 cycles at 95 °C for 15 sec and 60 °C for 1 min) on an Applied Biosystems 7300 Real-Time PCR System (Thermo Fisher Scientific, Applied Biosystems, Inc., Waltham, MA, USA). Data were normalised to β-actin expression (endogenous control), analysed using the cycle quantification value (Cq), and presented as 2^−ΔCq^. ΔCq is the difference between the Cq of the target gene (Cqt) and the reference gene (Cqr) (ΔCq = Cqt – Cqr) [47,48].

### 4.7. The CD3 Ζ Chain (CD247) Analysis in T Lymphocytes

Analysis of intracellular CD247 (TCRζ and CD3ζ) expressions was performed on fresh PBMC from CLL patients. PBMC were stained (20 min at RT) with mouse anti-human CD3 PE-Cy5 (Clone HI98, Cat No.: 555341) supplied by BD Biosciences. Next, cells were fixed with Cytofix/Cytoperm and permeabilized with a perm/wash buffer according to the manufacturer’s protocol. Then, an intracellular staining with FITC mouse anti-human CD247 antibody (Clone 6B10.2, Cat No.: 644104; BioLegend, San Diego, CA, USA) (30 min at 4 °C in the dark) was performed, and finally, cells were washed twice in PBS. Then, the sample was analysed using FACSCalibur (BD Biosciences). Kaluza 2.1.1 software was used for the data analysis. The results were expressed as the MFI of CD247 in CD3+ lymphocytes. Data were normalised to the FMO control and presented as ΔMFI. ΔMFI is the difference between the MFI of the specimen stained for CD3ζ and the MFI of cells unstained for CD3ζ (ΔMFI = MFI CD3ζ – MFI FMO).

### 4.8. Statistical Analysis

The statistical significance was calculated with either a Kruskal-Wallis test with Dunn correction or U Mann-Whitney test. Correlations were tested with the Spearman rank correlation coefficient. Kaplan Meier curves were used to plot time-to-treatment (TTT) and overall survival (OS) distributions among groups; the log-rank test was used for between-group comparisons and hazard ratios (HR) for quantification. Cox proportional hazard regression model was used for multivariable analysis. ZAP-70 is one of the most important and significant prognostic factors in CLL [49]. Therefore, ZAP-70 was used in the receiver operating characteristics (ROC) curve analysis. This analysis was used to calculate the appropriate M-MDSC percentage cut-off value so that it best distinguished ZAP-70-positive and ZAP-70-negative cases. The area under the curve (AUC) was also estimated. Differences were considered statistically significant with a *p*-value < 0.05. Statistical analysis was performed in Statistica 13 PL (StatSoft, Cracow, Poland). Graphs were prepared using GraphPad Prism version 5 (GraphPad Software, Inc., La Jolla, CA, USA) and Statistica 13 PL.

## 5. Conclusions

In conclusion, we have observed an increased population of M-MDSC cells in CLL patients. The close association with unfavourable prognostic markers (i.e., ZAP-70, CD38, 11q and 17p deletion, and IGHV mutational status) suggests a potential role of M-MDSC as a prognostic factor. In addition, the frequency of M-MDSC was increased in patients who required therapy. We have established an association between a high M-MDSC percentage on the one side and shorter time-to-treatment and overall survival on the other. IDO seems to be significantly involved in M-MDSC-mediated T cell suppression. Thus, we can suggest the adverse role of M-MDSC in CLL. Finally, M-MDSC seem to be an important new prognostic factor in CLL; as the method to quantify the M-MDSC percentages by flow cytometry is relatively easy and cheap, we propose performing it for every freshly diagnosed CLL case.

## Figures and Tables

**Table 1:** Characteristics of chronic lymphocytic leukaemia (CLL) patients at the time of diagnosis. Using 9.35% as a cut-off value (according to the receiver operating characteristic (ROC) analysis), the cohort was divided into two groups: monocytic myeloid-derived suppressor cell (M-MDSC)^low^ (less than 9.35% of M-MDSC) and M-MDSC^high^ (9.35% or more of M-MDSC) groups.

Variable	All Patients	M-MDSC^low^	M-MDSC^high^
No. of patients (%)	110	56 (50.1)	54 (49.9)
Sex			
Female (%)	60 (51.8)	26 (46.4)	34 (63.0)
Male (%)	50 (48.2)	30 (53.6)	20 (37.0)
Rai Stage			
0 (%)	56 (50.9)	33 (58.9)	23 (42.6)
I (%)	25 (22.7)	15 (26.8)	10 (18.5)
II (%)	17 (15.5)	6 (10.7)	11 (20.4)
III (%)	7 (6.4)	1 (1.8)	6 (11.1)
IV (%)	5 (4.5)	1 (1.8)	4 (7.4)
ZAP-70 (cut-off 20%)			
Positive (%)	38 (34.5)	15 (26.8)	23 (42.6)
Negative (%)	72 (65.5)	41 (73.2)	31 (57.4)
CD38 (cut-off 30%)			
Positive (%)	37 (33.6)	14 (55.3)	23(24.6)
Negative (%)	73 (66.4)	42 (44.7)	31 (75.4)
IGHV gene mutation status			
Mutated (%)	26 (23.6)	17 (30.4)	9 (16.7)
Unmutated (%)	19 (17.3)	6 (10.7)	13 (24.1)
Not available (%)	65 (59.1)	33 (58.9)	32 (59.2)
Cytogenetic abnormalities			
del(17p13.1) (%)	5 (4.5)	4 (7.1)	1 (1.9)
del(11q22.3) (%)	14 (12.7)	5 (8.9)	9 (16.6)
del(17p13.1) and del(11q22.3) (%)	2 (1.8)	1 (1.8)	1 (1.9)
Without del(17p13.1) and del(11q22.3) (%)	89 (81.0)	46 (82.2)	43 (79.6)
Patients requiring therapy (%)	26 (23.6)	7 (12.5)	19 (35.2)
Untreated patients (%)	84 (76.4)	49 (87.5)	35 (34.8)
No. of Deaths (%)	8 (7.3)	1 (1.8)	7 (12.9)
Age at diagnosis (years) *	65 (47–87)	66 (48–87)	64 (47–85)
WBC count (G/L) *	23.9 (10.1–298.4)	20.79 (10.72–26.8) ^a^	27.11 (10.1–298.4) ^a^
Lymphocyte count (G/L) *	17.86 (5.2–284.9)	16.39 (5.2–53.5) ^b^	22.7 (5.5–284.9) ^b^
LDH (IU/L) *	366 (178–618)	366 (246–618)	368 (178–579)
Haemoglobin (g/dL) *	13.9 (8.1–17.2)	14.0 (11.4–17.2)	13.8 (8.1–16.2)
Platelets (G/L) *	191 (49–414)	198 (90–414)	182 (142–394)
β2M (mg/dL) *	2.4 (1.3–5.4)	2.26 (1.4–4.2)	2.59 (1.3–5.4)
% CD19+/CD5+/ZAP-70+ cells *	13.2 (0.2–50.3)	11.97 (0.37–44.3)	14.5 (0.2–50.3)
% CD19+/CD5+/CD38+ cells *	6.9 (0.2–88.7)	4.99 (0.2–88.7)	9.38 (0.2–80.9)

IGHV, immunoglobulin heavy chain variable gene; WBC, white blood cell; LDH, lactate dehydrogenase; and β2M, β2-microglobulin. * median (range). ^a^ *p* < 0.05 and ^b^ *p* < 0.01.

**Table 2:** Univariate and multivariate analyses for time-to-treatment.

Variations	Univariate ^#^			Multivariate	
	Median TTT (months)	HR (95% CI)	*p*	HR (95% CI)	*p*
Age					
≥65 years	29	0.77 (0.35–1.70)	0.525		
<65 years	34				
ZAP-70					
≥20%	21	5.76 (2.48–13.38)	<0.0001	3.88 (1.43–10.47)	<0.01
<20%	35				
CD38					
≥30%	20	4.53 (1.99–10.30)	<0.01	1.64 (0.60–4.50)	0.333
<30%	34				
Β2M					
≥3.5 mg/dL	15	4.96 (2.24–10.97)	<0.0001	4.29 (2.01–11.16)	0.085
<3.5 mg/dL	36				
del(17p13.1) or del(11q22.3)					
Positive	20	3.22 (1.44–7.19)	<0.01	2.59 (1.12–6.02)	<0.05
Negative	32				
LDH					
≥ULN *	29	2.29 (1.10–4.76)	0.155		
<ULN	34				
*IGHV* mutation status^#^					
Unmutated	40	4.04 (1.27–12.80)	0.056		
Mutated	44				
M-MDSC					
≥9.35%	26	0.31 (0.13–0.72)	<0.01	0.39 (0.16–0.96)	<0.05
<9.35%	40				

TTT, time-to-treatment; β2M, β2 microglobulin; *IGHV*, immunoglobulin heavy chain variable gene; LDH, lactate dehydrogenase; and ULN, upper limit of normal. * The ULN of the LDH in this study was 250 IU/L. HR, hazard ratio and 95% CI: 95% confidence interval. ^#^ One hundred and ten patients were selected for the analysis. However, the *IGHV* mutational status was accessible for 45 participants of this study. Only variables with *p* < 0.05 in the univariate analysis were added to the multivariate analysis.

**Table 3:** Univariate and multivariate analyses for the overall survival.

Variations	Univariate ^#^			Multivariate	
	Median OS (months)	HR (95% CI)	*p*	HR (95% CI)	*p*
≥65 years	36	1.46 (0.34–6.13)	0.603		
<65 years	40				
ZAP-70					
≥20%	35	1.21 (0.28–5.05)	<0.01	0.39 (0.08–1.83)	0.238
<20%	41				
CD38					
≥30%	37	2.00 (0.50–8.01)	0.326		
<30%	38				
Β2M					
≥3.5 mg/dL	31	10.26 (2.06–50.89)	<0.01	10.36 (1.85–58.20)	<0.01
<3.5 mg/dL	40				
del(17p13.1) or del(11q22.3)					
Positive	33	4.15 (1.04–16.63)	<0.01	3.35 (0.82–13.80)	0.092
Negative	43				
LDH					
≥ULN *	32	0.41 (0.09–1.73)	0.22		
<ULN	41				
M-MDSC					
≥9.35%	33	0.22 (0.05–0.86)	<0.05	0.18 (0.02–1.44)	0.107
<9.35%	45				

OS, overall survival; β2M, β2 microglobulin; LDH, lactate dehydrogenase; and ULN, upper limit of normal. * The ULN of LDH in this study was 250 IU/L. HR, hazard ratio and 95% CI: 95% confidence interval. ^#^ One hundred and ten patients were selected for the analysis. Only variables in the univariate analysis with a *p* < 0.05 were added to the multivariate analysis.

**Table 4:** Intracellular IDO, Arg1, NOS2, IL-10, and TGF-β1 expressions by M-MDSC.

Variable	Unit	HV (*n* = 20) Median (IQR)	CLL (*n* = 60) Median (IQR)	*p*
IDO	%	2.32 (1.40–4.42)	60.51 (19.77–79.46)	<0.0001
	ΔMFI	214.6 (167.9–329.6)	350.0 (295.6–475.3)	<0.05
Arg1	%	51.69 (16.36–65.33)	86.19 (70.64-99.43)	<0.0001
	ΔMFI	359.8 (317.4–597.9)	608.5 (390.7–770.6)	<0.05
NOS2	%	21.64 (12.23–42.30)	70.77 (47.04–92.08)	<0.0001
	ΔMFI	255.7 (199.1–319.7)	468.0 (215.8–655.7)	<0.01
IL-10	%	0.66 (0.12–1.64)	27.34 (16.3–71.03)	<0.0001
	ΔMFI	275.3 (195.2–323.6)	390.0 (328.1–477.5)	<0.01
TGF-β	%	1.44 (0.70–4.64)	49.93 (22.17–81.29)	<0.0001
	ΔMFI	326.2 (229.0–397.7)	430.3 (332.3–553.5)	<0.01

MFI—median fluorescence intensity, IQR—interquartile range, and the *p*-value was calculated using the U Mann-Whitney test. HV: healthy volunteers, IDO: indoleamine 2,3-dioxygenase, Arg1: arginase 1, NOS2: nitric oxide synthase, IL-10: interleukin-10, and TGF-β: transforming growth factor beta.

## Supplementary Materials

The following are available online at https://www.mdpi.com/2072-6694/12/9/2614/s1: Table S1: The detailed configuration of the cytometers.
