# Supplementary material for: High M-MDSC Percentage as a Negative Prognostic Factor in Chronic Lymphocytic Leukaemia
Source: Cancers (Basel). 2020 Sep 14;12(9):2614. doi: 10.3390/cancers12092614 (PMC7563618; doi:10.3390/cancers12092614)
Supplement: Supplementary file 1 [file cancers-12-02614-s001.pdf]

Supplementary Materials: High M-MDSC Percentage as a Negative Prognostic Factor in Chronic Lymphocytic Leukaemia

Michał Zarobkiewicz, Wioleta Kowalska, Sylwia Chocholska, Waldemar Tomczak, Agata Szymańska, Izabela Morawska, Agnieszka Wojciechowska and Agnieszka Bojarska-Junak

Table S1. The detailed configuration of cytometers.

<table>
<tr><th colspan="4">BD FACS Canto II</th></tr>
<tr><th>No</th><th>Laser</th><th>Filter setup</th><th>Detector name</th></tr>
<tr><td>1</td><td rowspan="2">405</td><td>450/50</td><td>PacificBlue</td></tr>
<tr><td>2</td><td>510/50</td><td>AmCyan</td></tr>
<tr><td>3</td><td rowspan="4">488</td><td>530/30</td><td>FITC</td></tr>
<tr><td>4</td><td>585/42</td><td>PE</td></tr>
<tr><td>5</td><td>670LP mirror</td><td>PerCp-Cy5.5</td></tr>
<tr><td>6</td><td>780/60</td><td>PE-Cy7</td></tr>
<tr><td>7</td><td rowspan="2">633</td><td>660/20</td><td>APC</td></tr>
<tr><td>8</td><td>780/60</td><td>APC-Cy7</td></tr>
<tr><th colspan="4">BD FACS Aria IIu</th></tr>
<tr><th>No</th><th>Laser</th><th>Filter setup</th><th>Detector name</th></tr>
<tr><td>1</td><td rowspan="5">488</td><td>530/30</td><td>FITC</td></tr>
<tr><td>2</td><td>576/26</td><td>PE</td></tr>
<tr><td>3</td><td>610/20</td><td>PE-TexasRed</td></tr>
<tr><td>4</td><td>695/40</td><td>PerCp-Cy5.5</td></tr>
<tr><td>5</td><td>780/60</td><td>PE-Cy7</td></tr>
<tr><td>6</td><td rowspan="2">633</td><td>660/20</td><td>APC</td></tr>
<tr><td>7</td><td>780/60</td><td>APC-Cy7</td></tr>
</table>

© 2020 by the authors. Licensee MDPI, Basel, Switzerland. This article is an open access article distributed under the terms and conditions of the Creative Commons Attribution (CC BY) license (http://creativecommons.org/licenses/by/4.0/).
